# Protocol of randomized control trial for effectiveness of angiotensin receptor blockers on blood pressure control among euvolemic hypertensive hemodialysis patients

**DOI:** 10.1097/MD.0000000000006198

**Published:** 2017-04-07

**Authors:** Raja Ahsan Aftab, Amer Hayat Khan, Syed Azhar Syed Sulaiman, Tahir Mehmood Khan, Azreen Syazril Adnan

**Affiliations:** a Discipline of Clinical Pharmacy, School of Pharmaceutical Sciences, Universiti Sains Malaysia, Penang; b CKD Resource Centre, School of Medical Sciences, Universiti Sains Malaysia, Kubang Kerian, Kelantan; c School of Pharmacy, Monash University, Jalan Lagoon Selatan, Bandar Sunway, Selangor, Malaysia.

**Keywords:** hemodialysis, hypertension, losartan, postdialysis euvolemic hypertension, randomized control trial

## Abstract

**Introduction:**

Volume overload and the renin–aldosterone–angiotensin system (RAAS) are 2 major factors contributing to hypertension (HTN) among hemodialysis (HD) patients. Although volume-dependent components of HTN can be corrected by appropriate volume removal, a proportion of HD patients experience elevated blood pressure (BP) despite achieving euvolemic and ideal dry weight.

**Method and analysis:**

A single center, prospective, randomized, parallel design, single-blind trial will be conducted in the Malaysian state of Kelantan among postdialysis euvolemic hypertensive patients that are on regular dialysis at least 3 times a week. The primary outcome of the trial will be to note the effectiveness of losartan (RAAS inhibitor) in reducing systolic BP < 140 mm Hg compared to standard non-RAAS-inhibitor antihypertensive therapy. The secondary outcome will be to look at all causes of mortality. A body composition monitor (BCM) will be used to assess postdialysis volume and dry weight. Postdialysis euvolemic patients that have systolic BP > 140 mm Hg will be randomized using Covariate Adaptive Randomization to standard or treatment arm. Participants in the treatment arm will be given 50 mg of losartan once daily except on dialysis days, whereas the standard arm patients will be prescribed non-RAAS antihypertensive agents. The study participants will be followed for a period of 12 months. A Wilcoxon statistical test will be performed to note the difference in BP from baseline up to 12 months using Statistical Package for the Social Sciences (SPSS) 20.

**Ethical and trial registration:**

The study protocols are approved from the Ethical and Research Committee of the Universiti Sains Malaysia (USM/JEPeM/15050173). The trial is registered under the Australia New Zealand Clinical Trial Registry (ACTRN12615001322527). The trial was registered on 2/12/2015 and the 1st patient was enrolled on 10/12/2015. The trial was formally initiated on 16/02/2016.

**Conclusion:**

Management of HTN among HD patients requires understanding the primary cause of HTN and treating accordingly. The current trial is an attempt to reduce BP among postdialysis euvolemic but hypertensive patients.

## 1 Introduction

Chronic kidney disease (CKD) has long been identified as a risk factor for cardiovascular disease and other complications, whereas the number of kidney failure patients treated with hemodialysis (HD) has drastically increased in the last decade. In 2009, more than 570,000 people in the United States were classified as having end-stage renal disease (ESRD), including nearly 400,000 dialysis patients and over 17,000 transplant recipients.^[1,2]^ The rise in incidence of ESRD is attributed to an aging populace and increases in hypertension (HTN), diabetes, and obesity within the US population.^[3]^

In Malaysia, the 21st Report of the Malaysian Dialysis and Transplant Registry 2013 stated that 5491 new HD cases were registered, representing an acceptance rate of 15 per million, while new peritoneal dialysis cases totaled 731, representing an acceptance rate of 25 per million. The total number of HD and peritoneal dialysis patients in 2013 increased to 28,822 and 2815, respectively. Geographically, the dialysis rate exceeded 100 per million for all states in Malaysia in 2013 with the highest rates reported in Pulau Pinang (303 pmp) and the lowest in Perlis (104 pmp). Over the last 10 years, the ratio of male to female incident and prevalent dialysis patients has remained the same, at about 55:45.^[4]^

Up to 90% of hemodialysis patients are hypertensive. Studies aimed at elucidating the pathophysiology of HTN HD patients concluded that 90% of cases resulted from sodium and volume overload (volume-dependent), while the majority of the remaining had elevated renin activity (renin-dependent), resulting in high blood pressure (BP) during HD.^[5]^ The renin–angiotensin–aldosterone system (RAAS) is a signaling pathway responsible for regulating the body's BP. Stimulated by low BP or certain nerve impulses, the kidneys release an enzyme called renin that undergoes various transformations, finally resulting in angiotensin II,^[6]^ which not only causes blood vessels to narrow (vasoconstriction), but also simultaneously stimulates the secretion of vasopressin (also called AVP). In this way, the overall volume of blood in the body is increased: more blood is pumped through constricted arteries, which increases the pressure exerted on the artery walls – so increasing the BP. Interestingly, the RAAS is activated in HD patients by the fact that renin is increased with HD ultrafiltration leading to HTN.^[7]^

Adequate extracellular volume control remains one of the principle goals for renal replacement therapies. Fluid balance is an integral part of HD to prevent under- or overhydration at the end of HD session, both of which are associated with cardiovascular and other complications.^[8]^ Hence, the correct quantification of extracellular fluid is essential for HD patients. A bioimpedence spectroscopy (a body composition monitor [BCM] manufactured by Fresenius Medical Care) allows quantification of extracellular fluid by comparison with a healthy population. A number of studies indicate that a BCM is an objective and reliable tool to assess the clinical dry weight and estimate extracellular fluid.^[9–12]^

The activation of the RAAS leads to narrowing of the lumen of blood vessels thus leading to a rise in BP even if the patient is euvolemic. Considering the importance of the RAAS in euvolemic hypertensive patients, the role of drugs blocking the RAAS needs further investigation.

### 1.1 Rationale of this study

In the past, emphasis has often been placed on overall BP reduction of among HD patients. However, a “one-size-fits-all” approach for BP management may not be appropriate for all the patients on HD.^[13]^ The renin angiotensin system is activated in HD patients by the fact that renin is increased with HD ultrafiltration, leading to HTN. This makes the RAAS a potential target for antihypertensive drugs.^[14]^ Normally, volume overload and elevated BP suppress the renin system. Since this feedback mechanism is incomplete in CKD patients, they often have high BP with normal or elevated renin levels.^[15]^ Literature suggests that renin levels may be twice as high in hypertensive HD patients compared to normal, with a study reporting plasma renin activity increasing from 2.3 ± 0.5 ng/mL/h just before the initiation of HD to 6.5 ± 1.3 ng/mL/h over an 8- to 10-year period among hypertensive HD patients.^[15,16]^ This finding suggests that renin secretion continues even after complete decline of kidney function.

Fluid balance is an integral part of HD, and problems with it are associated with cardiovascular and other complications.^[17]^ The majority of fluid removal and maintaining an ideal hydration status is looked at by considering a patient's dry weight. However, dry weight is clinically determined by the lowest weight a patient can tolerate without intradialytic symptoms and hypotension in the absence of fluid overload. A bioimpedence device such as a BCM allows quantification of excess extracellular volume by comparison with a healthy population, thereby providing us with a more reliable measure of dry weight and patient hydration status.^[18]^ Importantly, volume-dependent components of HTN can be corrected by appropriate volume removal, though a proportion of HD patients experience elevated BP despite achieving euvolemic and ideal dry weight.^[19]^

Since there is a constant volume variation during HD sessions, there is a strong possibility that the RAAS will be activated, thereby making these patients a potential target of drugs blocking the RAAS. Hence, the current study is based on role of angiotensin receptor blockers (ARBs) in managing HTN among euvolemic HD patients.

### 1.2 Rationale for intervention

ARBs and angiotensin-converting enzyme inhibitors are similar and both are often used during clinical practice for ESRD patients. An observational study suggests that an ARB in combination with another antihypertensive medication (but not an angiotensin-converting enzyme inhibitor) may have a beneficial effect on cardiovascular mortality among ESRD patients on dialysis.^[20,21]^ Based on cost-effectiveness and availability, and the opinion of a panel of experts, a decision was taken to use an ARB (losartan) for the intervention group.

## 2 Method

### 2.1 Trial design

The current study will be a single-center, prospective, randomized, parallel-design, single-blind trial that would be conducted in the Malaysian state of Kelantan at a CKD resource center of the Hospital Universiti Sains Malaysia (HUSM) and its associated dialysis centers. The study protocols are approved by the Ethical and Research Committee of Universiti Sains Malaysia (USM/JEPeM/15050173), and the trial is registered under Australia New Zealand Clinical Trial Registry (ACTRN12615001322527). The study purpose will be explained, and patients’ consent will be obtained before enrolling by the principle investigator.

### 2.2 Eligibility criteria

Euvolemic patients with a postdialysis BP of more than 140/90 mm Hg will be included in the study. On the basis of expert opinion from nephrologists, patients aged between 30 and 80 will be included. The other eligibility criteria are: patients undergoing dialysis for at least 12 months, 2 to 3 HD sessions per week, and willingness to participate.

### 2.3 Exclusion criteria

The exclusion criteria are as follows: patients with amputations; patients with neoplasm; patients with cystic kidneys; patients unwilling to participate in the study; patients already on ARBs; and patients with symptomatic hypotension or systolic BP less than 110 mm Hg.

### 2.4 Prescreening

The current trial is a 2-phase study. Phase 1 is the prescreening procedure, including prospectively assessing the hydration status of hypertensive patients undergoing HD. After assessing the dry weight with a BCM, all efforts will be made to ensure the patient achieves the same dry weight at the end of their dialysis sessions. Patients that are still hypertensive (>140 mm Hg) 30 minutes after the dialysis sessions will undergo a volume assessment with a BCM device.

Patients that will be assessed as euvolemic by a BCM in 3 consecutive dialysis sessions but are still hypertensive (>140/90 mm Hg) despite achieving dry weight will be included in the trial. Since the purpose of phase 1 is to identify the euvolemic hypertensive population, it will be these patients that will be asked to join a randomized control trial (phase 2). A regular BCM assessment for dry weight will be done to ensure patients attain their specific dry weight and euvolemic states.

### 2.5 Randomization of participants

Covariate adaptive randomization will be used to assign all prescreened participants that agree to participate in the study to the treatment or standard arm. Covariate adaptive randomization has an advantage over other randomization techniques in that it is able to achieve balance over a large number of covariates when the sample size is small to medium.^[22]^ The Taves covariate adaptive randomization method will be used for randomization to allow for the examination of previous participant group assignments in order to make a case-by-case decision on group assignment for each individual who enrol in the study. Using covariate adaptive randomization, patients will be assigned to the treatment or control arm. Based on expert opinion and preexisting literature, the covariates considered for this study will be: age, gender, diabetes, and year of dialysis. In order to avoid bias, randomization will be done by a computer programme, hence minimizing the risk of selection biasness and avoiding any influence of the researcher, the prescriber, or the participant on selection of group or medication. It is important to mention however that Scott et al^[23]^ argued that predictability and bias of randomization is true for all methods and so should not be overly penalized.

### 2.6 Washout period

The purpose of the washout period is to eliminate previously given hypertensive medication. Since all enrolled patients will be hypertensive, care has to be taken not to deprive patients of antihypertensive therapy. During the washout period patients, in consultation with a cardiologist and a nephrologist, will be maintained on other hypertensive medication (except RAAS inhibitors) to maintain their BP and to avoid any bias in the study. On the completion of the washout period, the patients will enter the regular trial phase.

### 2.7 Standard and intervention arms

Both the standard and intervention arms will be randomized with equal numbers of patients. The standard-arm patients will receive antihypertensive therapy except RAAS inhibitors that included calcium-channel blockers, diuretics, alpha- and beta-blockers, whereas the intervention-arm patients will be prescribed ARB (losartan) alone or in combination with standard antihypertensive pharmacotherapy. In both study arms, patients will be allowed multiple antihypertensive pharmacotherapies to control BP, after discussion with a panel of experts. However, this will only be done where entirely necessary to avoid any deterioration to a patient's health. Finally, study intervention (losartan) and antihypertensive medication will be given to the patients on a weekly basis by the principle investigator. A member of patient's family will be consented to monitor a patient's adherence at home whereas a pill check will be performed once a week. Only the study participants will be blinded during the course of study.

### 2.8 Study end point

A medication dose of 50 mg losartan per day for 3 weeks will be initiated as a test dose to note any incidence of hypotension. If there is no hypotensive event, patients will continue with 50 mg daily every day except dialysis days. The dose could be a titrated dose up to 100 mg/day, depending upon expert opinion and clinical scenario. As for treatment group, the dose titration would be initiated with losartan 50 to 100 mg followed by non-RAAS antihypertensive agents. As for standard group, the choice of dose titration would be left to expert opinion. The primary end point will be achieved by having a postdialysis BP of <140/90 mm Hg and maintaining this for 4 weeks. The secondary end point will be looking at all causes of mortality.

### 2.9 Probability, severity, and intensity of adverse drug reactions (ADR)

All records of commonly occurring ADRs will be maintained throughout the study period. The probability of ADR will be measured using a Naranjo scale,^[24]^ whereas the severity and intensity of ADRs will be evaluated by Hartwig scale and Schumock scale, respectively.^[25]^

### 2.10 Data handling and record keeping

All BCM analysis and initial data collection will be performed by the chief investigator himself. Proper patient IDs will be given to each patient to use for future reference throughout the study. Since BP readings and other important clinical parameters will be noted in every dialysis session, a data collection logbook will be used to note pre-, intra-, and postdialysis BP and other information. All data will be collected by the principle investigator. Once in 2 months, all enrolled patients will be visited in HUSM, as per the study protocol. During this visit, patients will undergo routine examinations and routine blood test (complete blood picture) by nephrologists. All data collection forms, incidence reporting forms, and patient logbooks will be kept by the principle investigator and will be used for data analysis. All data will be kept highly confidential to minimize any bias.

### 2.11 Potential risk to subjects

One of the main potential risks among the study subjects is uncontrolled HTN or hypotension. Patients will be monitored closely to minimize risks. Any patient during the trial with a systolic BP >200 or <110 mm Hg over 3 dialysis sessions will be assessed by the physician in charge, and a decision will be made regarding such patients upon discussion with a panel of experts. Regular blood samples will be taken to avoid any episode of ARB-associated hyperkalemia and other blood abnormalities. Thorough records will be maintained for this purpose. Other minor side effects are common with all antihypertensive medication, including dry cough, nausea, vomiting, dizziness, and headache. Participation in the study is voluntary and all participants will have the right to withdraw from the study. In case of any study-related injury or where a nephrologist feels that the patient might be at risk of study-related injury, the patient will be withdrawn from the study immediately and treated at HUSM. Similarly in case of any ADR developed during the study, the patient will be referred to and treated at HUSM.

### 2.12 Sample size

The sample size for the current study was based on a statistical superiority trial (continuous data) design of a randomized control trial. To verify that a new treatment is more effective than a standard treatment from a statistical point of view or from a clinical point of view, its corresponding null hypothesis is that the new treatment is not more efficacious than the control treatment by a statistically/clinically relevant amount. Based on the nature of the relevant amount, a superiority design contains statistical superiority trials and clinical superiority trials.^[26]^

Where N is the size per group, *p* the response rate of standard treatment group; *p* 0 the response rate of new drug treatment group; *z* the standard normal deviate for a 1 or 2 sided *x*; *d* the real difference between 2 treatment effect; a clinically acceptable margin; and S is the Polled standard deviation of both comparison groups. 
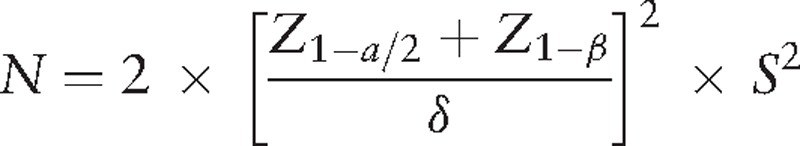


Calculating the sample size using the equation above: 
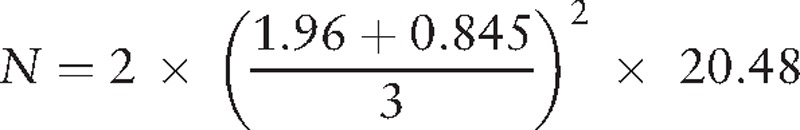


N = 35

The sample size calculated from statistical superiority for randomized control trial is 35 for each arm of the treatment, so altogether 70 euvolemic hypertensive patients should be recruited for the current study. Since a 25% dropout rate has to be anticipated, the final total was 88:44 in each arm.

### 2.13 Statistical analysis

**Figure 1:**
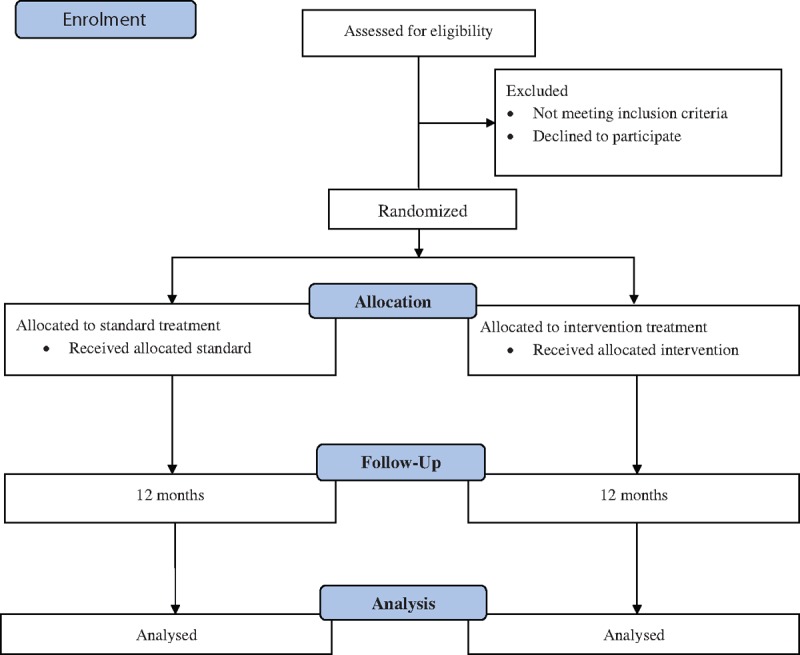
Study flow diagram.

Results will be expressed as mean or percentage. Comparisons between treatment groups will be made by using a Wilcoxon test after adjustment for the dynamic stratification variables (age, sex, years on dialysis, and diabetes). Cohen d test will be applied to note the effect size. In addition, linear and logistic regression will be applied to note any influence of patient characteristics on treatment outcome. This data will be presented as hazard ratios and 95% confidence intervals. Statistical significance will be set at *P* less than 0.05. All statistical calculations will be performed using Statistical Package for the Social Sciences (SPSS) version 20. Figure 1 provides details of study flow diagram.

## 3 Discussion

The management of euvolemic HTN among HD patients has often been neglected. The burden of cardiovascular morbidity and mortality among HD patients is often associated with elevated BP.^[27]^ Although it is not the primary outcome of our trial, the ultimate goal of our intervention is to reduce mortality among patients undergoing HD. This study addresses HTN management in postdialysis euvolemic patients.

Interventions such as lipid lowering, dialysis prescription modification, and mineral metabolism modification have been assessed in multiple randomized control trials and systematic reviews, but there is no clear evidence that any of these approaches reduces mortality among HD patients.^[28–31]^ Meta-analysis suggests that treatment with agents lowering BP tends to reduce cardiovascular mortality among patients undergoing HD.^[32]^ Studies suggest that agents blocking the RAAS, calcium-channel blockers, and β-blockers are suitable for use in HD patients and should be the 1st line of therapy. ACE inhibitors have shown almost similar efficacy as ARBs in the general population. However, ARBs have shown better results than ACE inhibitors among HD patients.^[20,21]^

Guidelines from the National Kidney Foundation of Malaysia recommend a predialysis BP of <140/90 mm Hg and postdialysis BP of <130/80 mm Hg as a targeted BP among HD patients. However, there are some concerns regarding the targeted BP, since most of the data are manipulated from observational studies from non-ESRD patients, so targeted BP among HD patients remains unclear.^[33]^

Volume overload is an important contributor in the pathogenesis of high BP among HD patients. Results from studies indicate that volume control greatly reduces BP in patients undergoing HD.^[34]^ Hence, maintenance of volume control is critical in achieving BP targets. Other factors including increased sympathetic activity and activating the RAAS have been shown to affect BP among HD patients. The RAAS is activated in HD patients because renin is increased by ultrafiltration. Moreover, infusion of angiotensin II antagonist improves BP control. Patients whose BP is not controlled by maintaining dry weight have shown to have increased plasma renin activity. A dramatic improvement in BP is noticed after interruption of the renin angiotensin axis by bilateral nephrectomy.^[35,36]^

Randomized control trials are the most rigorous way of determining the cause-effect relationship between treatment and outcome. Nonrandomized control trials can also be used, but the possibility of association caused by a 3rd factor cannot be ruled out.^[37]^

From the above discussion, we know that HTN is common in HD patients and is closely related to cardiovascular morbidity and mortality. In HD, although HTN is multifactorial, it is mainly influenced by volume overload and RAAS. Maintenance of dry weight is essential in achieving a euvolemic state at the end of dialysis. Achieving targeted dry weight and a euvolemic state should result in an ideal BP range, but some HD patients, despite achieving targeted dry weight and a euvolemic state, are still hypertensive. A strong possibility exists that these patients are hypertensive under the RAAS.

Keeping in mind all of these factors, a randomized control trial was planned among postdialysis patients that despite being euvolemic was still hypertensive. The trial included a standard and an intervention arm, where the standard arm received non-RAAS antihypertensives including calcium-channel blockers, α- and β-blockers, and diuretics that are commonly prescribed in routine clinical practice, and the intervention arm received an ARB (losartan) alone or in combination with standard antihypertensive pharmacotherapy.

Often in the past emphasis was put on overall BP reduction in HD patients. However, the one-size-fits-all approach for BP control among HD patients may not be appropriate. Similarly, management of euvolemic HTN requires treatment as a separate entity. Studies have been performed in assessing BP control with multiple antihypertensive agents among HD patients. However, to our knowledge, this is a 1st attempt to study the effect of medication blocking RAAS (losartan) on euvolemic hypertensive patients.

The current study is a multicenter study planned to be carried out in the state of Kelantan of Malaysia. Although Malaysia is a multiethnic country, the state of Kelantan is mostly inhabitant by the local Malay race. Thereby, the majority of patients enrolled in the trial will be Malays. Since the prevalence of HD patients has doubled in Malaysia in the last decade, it is important to know the reasons behind this. On the other hand, efforts to manage these patients and improve their quality of life must not be neglected.

### 3.1 Limitations of study

A small sample size is one of the main limitations of this study. Future studies should consider addressing the similar study question in a larger and diverse population. Although Malaysia is a multiethnic country, the majority of study participants will be Malay race. Therefore, the results of this study cannot be generalized for the whole population of Malaysia. Keeping in mind the principle of genetic influence, it might be possible that other ethnic groups, for example, Chinese or Indians, might have different outcomes to Malays. Accurate BP measurement is fundamental to clinical practice and research, yet there is no consensus on which BP measurements to use and define HTN in HD patients, as studies indicate that predialysis or postdialysis BP readings that are routinely done may overestimate BP.^[38]^ A similar difference in the method for measuring the BP also exists, which is whether the BP should be read when the patient is in a supine or sitting position, though the literature reports that it makes no difference. Hence, there is no consensus over the optimal method of BP measurement among HD patients.^[39]^ Nonpharmacological interventions such as diet and fluid intake also play their role in HTN management. Educating the study participants regarding the importance of balance diet will be part of this current trial, as nonadherence to diet regimen or strict diet control may influence BP and so may affect study outcomes. A close family member will be asked to monitor diet regimen and medication adherence.

## Acknowledgments

The authors thank Institute of post graduate studies (IPS) of Universiti Sains Malaysia for providing fellowship during the course of doctoral studies of Mr Raja Ahsan Aftab.
